# Magical Mathematical Formulas for Nanoboxes

**DOI:** 10.1186/s11671-021-03472-8

**Published:** 2021-03-01

**Authors:** Forrest H. Kaatz, Adhemar Bultheel

**Affiliations:** 1 Mesalands Community College, 911 South 10th Street, Tucumcari, NM 88401 USA; 2 Department of Computer Science, KU Leuven, Celestijnenlaan 200A, 3001 Heverlee, Belgium

**Keywords:** Nanobox, Nanocage, Nanoframe, Coordination, Magic numbers, Dispersion

## Abstract

Hollow nanostructures are at the forefront of many scientific endeavors. These consist of nanoboxes, nanocages, nanoframes, and nanotubes. We examine the mathematics of atomic coordination in nanoboxes. Such structures consist of a hollow box with *n* shells and *t* outer layers. The magical formulas we derive depend on both *n* and *t*. We find that nanoboxes with *t* = 2  or  3, or walls with only a few layers generally have bulk coordinated atoms. The benefits of low-coordination in nanostructures is shown to only occur when the wall thickness is much thinner than normally synthesized. The case where *t* = 1 is unique, and has distinct magic formulas. Such low-coordinated nanoboxes are of interest for a myriad variety of applications, including batteries, fuel cells, plasmonic, catalytic and biomedical uses. Given these formulas, it is possible to determine the surface dispersion of the nanoboxes. We expect these formulas to be useful in understanding how the atomic coordination varies with *n* and *t* within a nanobox.

## Introduction

Nanoboxes were originally synthesized circa 2002 [1, 2]. A nanobox is distinct from a nanocage in that the latter has porous walls. Also, both are distinct from a nanoframe, in that the nanoframe is a structure (frame) consisting of the low-coordinated outline of the cluster. Such anisotropic, polyhedral structures may be created from galvanic displacement reactions [3, 4]

1 $$\begin{array}{l}\\ {\hbox{Anode}}: yA_{(s)}\rightarrow yA_{({{\rm aq}})}^{x+}+xye_{({{\rm aq}})}^{-} \\ {\hbox{Cathode}}: xB_{({{\rm aq}})}^{y+} + xye_{({{\rm aq}})}^{-} \rightarrow xB_{(s)} \\ {\hbox{Full\,Reaction}}: yA_{(s)}+xB_{({{\rm aq}})}^{y+} \rightarrow yA_{({{\rm aq}})}^{x+}+xB_{(s)} \\ \end{array}$$

where a nanocluster with metal *A* is sacrificially hollowed out by an aqueous solution of metal *B*, which has a higher reduction potential and creates the hollow solid of element *B*. Half reactions occur at the anode and cathode of an electrochemical cell, resulting in the full combined reaction as above [5]. In some instances, scientists have combined galvanic displacement with void formation via Kirkendall Fickian diffusion of metals and vacancies [6]. Models for this activity exist for specific cases and in situ electron microscopy experiments have been reported [7, 8]. Other synthetic methods include chemical etching [9], ion exchange [10], and metal–organic frameworks (MOFs) [11, 12]. A recent review of synthesis methods mentions that anisotropic clusters have yet to be made in the size region $2<D<20$ nm, hindering the progress of nanocage fabrication in this important size domain [13].

Such hollow structures have low coordination, making them of interest for batteries [12], fuel cells [14], plasmonic [15], catalytic [16], and biomedical applications [17]. Previous analysis shows that for catalytic applications, a coordination approach applies [18], while for energy storage, there are only some hints with density functional theory (DFT) results indicating that select facets are important [19]. We use a previously derived method from adjacency matrix analysis [20, 21] to discover the atomic coordination of a box with *n* shells and a wall thickness of *t* layers. This analysis shows that a nanobox with *t* = 2  or  3 has bulk coordination and as such the benefits of low coordination are present only for nanoboxes with thinner walls than generally believed necessary. The methods we use quantifies the atomic coordination through magic numbers and formulas for thirteen types of nanoboxes.

## Methods

Key to our analysis by coordination methods, is the creation of an adjacency matrix from the atomic coordinates of the nanobox. Such a matrix is created as follows. We define *i* and *j* as nearest neighbors, and separate them from the rest by requiring that the bond length $r_{ij} < r_c$ where $r_c$ is a threshold value, appropriate for the nanobox. The value for $r_c$ must be less than the distance for second nearest neighbors and varies with the crystal structure [21]. For bcc crystals, $r_c < 1.15\cdot r_{{\rm min}}$, where $r_{{\rm min}}$ is the smallest bond length. Thus,

2 $${\mathbf{A}}(i,j)=\left\{ \begin{array}{ll} 1 &{\quad}\hbox { if } r_{ij}< r_c \,\,\hbox { and } \,\,i\ne j\\ 0 &{\quad} \hbox {otherwise}\end{array}\right.$$

describes the adjacency matrix for the cluster, and

3 $${\mathbf{E}}(i,j)=\left\{ \begin{array}{ll} r_{ij} &{\quad}\hbox { if } \, r_{ij}< r_c \,\,\hbox { and }\,\, i\ne j\\ 0 &{\quad} \hbox {otherwise}\end{array}\right.$$

describes the Euclidean matrix for the box. We use the Euclidean matrix to determine the diameter, *D*, (nm) for the nanoboxes.

Since we create nearest neighbor adjacency matrices, we know the coordination number $\hbox {cn}_i$ of vertex *i* by summing the elements of ${{\mathbf{A}}}(i,:)$. Our structure consists of $n+1$ shells numbered 0, 1, ..., *n*, with *t* outer layers. Let $N_{{\rm cn}_i}(n,t)$ be the number of atoms with coordination $\hbox {cn}_i$ where $1\le \hbox{cn}_i\le \hbox{cn}_M$ with $\hbox {cn}_M$ the maximal coordination in the nanobox. Then the total number of atoms in the nanobox is given by

4 $$N_T(n,t) = \sum _{{\rm cn}_i=1}^{{\rm cn}_M}{N_{{\rm cn}_i}(n,t)}.$$

The surface atoms in the outer shell (or interior) of the nanobox, *n* have a set of bondings less than the bulk coordination. Thus the maximal coordination for surface atoms is $\hbox {cn}_s < \hbox{cn}_M$, and the number of surface atoms is

5 $$N_S(n,t) = \sum _{{\rm cn}_i=1}^{{\rm cn}_s}{N_{{\rm cn}_i}(n,t)}.$$

This holds if all the non-surface vertices have coordination larger than $\hbox {cn}_s$, which is true for all fcc, bcc, and hcp clusters. We determine the $N_{{\rm cn}_i}(n,t)$ by counting the columns of the adjacency matrix whose sum is $\hbox {cn}_i$. Note that our cluster coordinate algorithm is built by shells, so that each subsequent shell contains all the previous lower values of *n*. In addition, the number of bonds in the box is

6 $$N_{{\rm B}}(n,t) = \frac{1}{2}\sum _{{\rm cn}_i=1}^{{\rm cn}_M}{\hbox{cn}_i\cdot N_{{\rm cn}_i}(n,t)},$$

where $N_{{\rm B}}(n,t)$ is the number of bonds and $\hbox {cn}_M$ is the maximum coordination. The factor of 1/2 comes about because of the pairwise nearest neighbor bonding.

Since we know that these equations depend on *n*, *t* as a polynomial of degree at most 3, we can compute $N_{{\rm cn}_i}(n,t)$ for 4 consecutive values of *n*, say $n=n_0+j$, *j* = 0, 1, 2, 3. A simple interpolating polynomial will then give the polynomial coefficients. It has to be verified that by increasing $n_0$, which is usually equal to 1, the formulas do not change. If the formulas become stable from $n_0$ on, then they hold for all $n\ge n_0$. To get the exact rational coefficients, one needs to solve the Vandermonde system for the coefficients in exact arithmetic.

Note that in the magical formulas for nanoboxes we have that $n > t$ so that therefore contrary to any expectation, filling up the box by an appropriate choice of *t* will not re-create the original magic formulas for the complete solid clusters. These magic formulas are useful for modeling the mesoscale properties of clusters and boxes, or cages. Complete sets of formulas were originally derived for nineteen cluster types. In this manuscript, we derive magical formulas for thirteen types of nanoboxes.

In the magical formulas below, we find that bulk coordination may appear for either *t* = 2 or *t* = 3 layers of shell thickness. Most are for layers where *t* = 2; the exceptions are the fcc cube, the cuboctahedron, the icosahedron, and the bcc cube and truncated cube. In the latter, bulk coordination only appears for *t* = 3 layers. For the data below, the tables of the magical formulas are accompanied by a figure of a ‘half-box’ to show the interior of the nanoboxes. Alongside is a colorbar indicating the coordination and number of such in parentheses.

## Results and Discussion

In order to delineate the applicability of magic formulas, we outline how catalytic behavior may depend on coordination and such formulas. We define *G* as the size dependent Gibbs energy of the cluster. Because of adatoms being bonded to the outer shell atoms there is an increase in *G* that is called the adsorption energy and is denoted as $\Delta G$. This can be split up over different coordination types of the atoms on the outer shell bonding to adatoms. For example, a kink atom adds to the adsorption energy with an amount $\Delta G_{k}$. Similarly an edge atom adds $\Delta G_{e}$, while a facet atom contributes $\Delta G_f$ then [18]:

7 $$\Delta G=\sum _{o\in \{f,e,k\}} \Delta G_o N_o$$

where $N_o$ is the number of atoms in the outer shell of the indicated type. The total number of atoms in the outer shell bonded to adatoms is defined as $N_s=N_f+N_e+N_k$, resulting in:

8 $$\begin{aligned}\Delta G= & {} \Delta G_f\cdot (1-f_e-f_k)+\Delta G_e\cdot f_e+\Delta G_k\cdot f_k \\&\quad {\hbox{where}} \,\,f_o=N_o/N_s, \,\,o\in \{e,k\},\end{aligned}$$

with the Gibbs energy fraction expressed through the edge and kink sites which have explicit coordinations for specific structures. This demonstrates that magic formulas have a role in surface reactions, through edge and kink coordinations and their formulas. Note that Eq. (8) applies to adsorption to on-top sites, otherwise not all adatoms will be bonded to atoms in the outer shell. In such a model, the kink sites have magic formulas that are constant with the number of shells, *n*, edge sites have formulas that are linear with *n*, and facet sites have formulas that are quadratic with *n*. More specifically, the kink sites are the lowest coordinated formulas, the edge sites are the second lowest coordinated, and facet sites have cn = 8 for (100) facets and cn = 9 for (111) facets.

Two fundamental relationships on a per-particle basis can be applied. For the Gibbs energy and adsorption constant, $K_{{\rm a}}$, it holds:

9 $$K_{{\rm a}}={\exp } \left( -\frac{\Delta G}{RT}\right) ,$$

where *R* is the gas constant and *T* is the temperature in Kelvin. In addition, Brønsted–Evans–Polanyi relationships are widely used in homogeneous and heterogeneous catalysis [18, 22] using a relationship for reaction constants *k* and equilibrium constants *K* as follows:

10 $$k=gK^{\alpha },\quad 0< \alpha < 1,$$

where *g* and $\alpha$ (Polanyi parameter) are constants. The Polanyi parameter is unitless and a proper fraction, as given originally by Brønsted [23]. We then have:

11 $$k=k'_{a}{\exp } \Bigl (-\alpha \bigl ({f_n^{e}\cdot \chi _{_e}({D_n}) +f_n^{k}\cdot \chi _{_k}({D_n})}\bigr )\Bigr ),$$

where

12 $$\begin{aligned} &\chi _{_e}(D)=\frac{\Delta G_e(D)-\Delta G_f(D)}{RT},\\& \chi _{_k}(D)=\frac{\Delta G_k(D)-\Delta G_f(D)}{RT}, \end{aligned}$$

and

13 $$k'_{a}=g\exp \left( -\alpha \frac{\Delta G_f}{RT}\right) .$$

This analysis shows that determining a catalytic model necessitates a method of calculating the Gibbs energy. Known catalytic reactions such as the two-step and Langmuir–Hinshelwood mechanisms have been considered [24].

## FCC Nanoboxes

Face centered cubic structures are the most common form for nanoclusters and nanoboxes. This is the structure of the metals with interesting properties, such as the noble metals with plasmonic properties, and the catalytic precious metals. Since gold has a high reduction potential of 1.50 V (see Eq. 1) versus the standard hydrogen electrode (SHE) [5], it is one of the easiest metals to synthesize as a nanobox or nanocage. Gold nanoboxes or nanocages have been formed in cubic [1], cuboctahedron [25], icosahedron and decahedron [26], octahedron [27] and tetrahedron [28] shapes.

We can determine the approximate size of these nanoboxes by using a coordination approach for the nearest neighbor bond length *r*(cn) [29],

14 $$r(cn) = \frac{2r_{{\rm B}}}{\left( 1+\exp \left( \frac{12 - \langle cn \rangle _c)}{8\cdot \langle cn \rangle _c}\right) \right) }.$$

Here $r_{{\rm B}}$ is the bulk bond length for gold (0.2884 nm) and $\langle cn \rangle _c$ is the average coordination of the cluster. We find a linear relationship between *D* and *n*, the number of cluster shells, as shown in Table 1:

15 $$D(n)=a \cdot r_{{\rm B}} \cdot n + b.$$

We use nanoboxes with *t* = 3, as the formulas vary with *t*, and we wish to achieve some bulk coordination. For the calculation of *D*(*n*), we use the maximum distance between atoms in the cluster, derived from the Euclidean matrix. Note that *D*(*n*) is an empirical formula, derived from data (vary *n* and calculate *D*), and as such is not proven.

**Table 1:** Linear constants for *D*(*n*)

a	b	Nanocluster
2.0	− 0.0374	Au cuboctahedron

a	b	Nanobox *t* = 3
1.9700	− 0.0060873	Au cuboctahedron
2.4074	− 0.003519	Au cube
2.3902	− 0.37914	Au truncated cube
1.3980	− 0.0093924	Au octahedron
0.9888	− 0.021186	Au tetrahedron
1.4821	− 0.003994	Au icosahedron
1.6280	− 0.0068481	Au decahedron

These relationships produce diameters in agreement with other data, from DFT. For the solid cuboctahedra with *N* equal to 55, 561, and 923 we get diameters of 1.12 nm, 2.85 nm, and 3.43 nm. This compares favorably with published DFT results for 55 atoms of 1.1 nm [30], for 561 atoms, 2.7 nm [31], and for 923 atoms, 3.5 nm [30]. The magical formulas for some fcc nanoboxes are tabulated below (Tables 2, 3, 4, 5, 6, 7, 8).

**Table 2:** Magic formulas for the fcc cube (even layers)
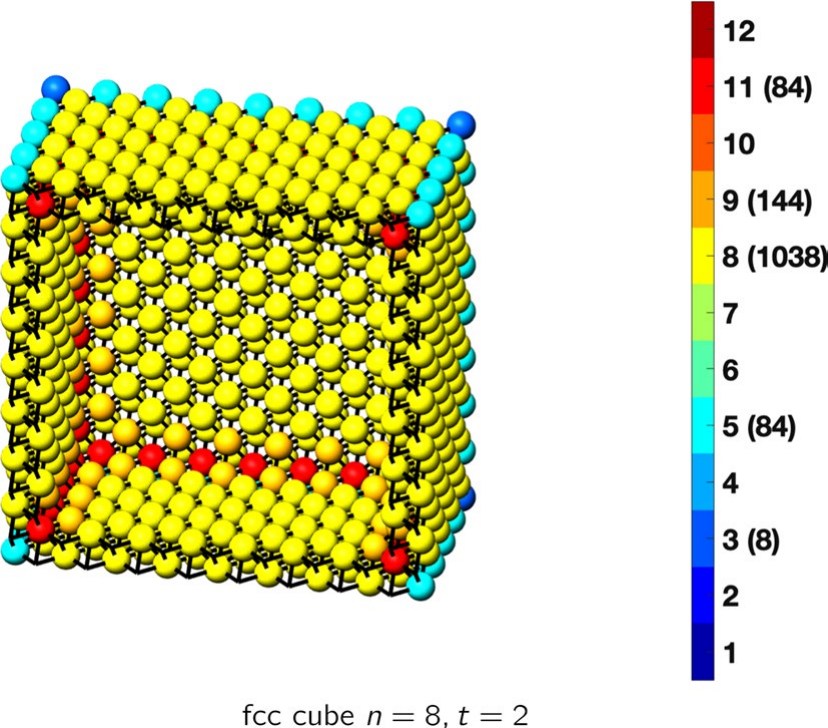

fcc cube	
Atoms	$(12t)n^2+(-12t^2+12t)n+(4t^3-6t^2+3t), \,n>t\ge 4$
Bonds	$(72t-48)n^2+(-72t^2+120t-36)n+(24t^3-60t^2+36t-12),\,n>t\ge 4$
cn = 3	$8,\,n>t\ge 2$
cn = 5	$12n-12,\,n>t\ge 2$
cn = 8	$24n^2+(-24t-24)n+(12t^2+12t+6),\,n>t\ge 2$
cn = 9	$24n +(-24t),\,n>t\ge 2$
cn = 11	$12n+(-12t+12),\,n>t\ge 2$
cn = 12	$(12t-24)n^2+(-12t^2+36t-24)n+(4t^3-18t^2+27t-14),\,n>t\ge 4$

**Table 3:** Magic formulas for the fcc cube (odd layers)
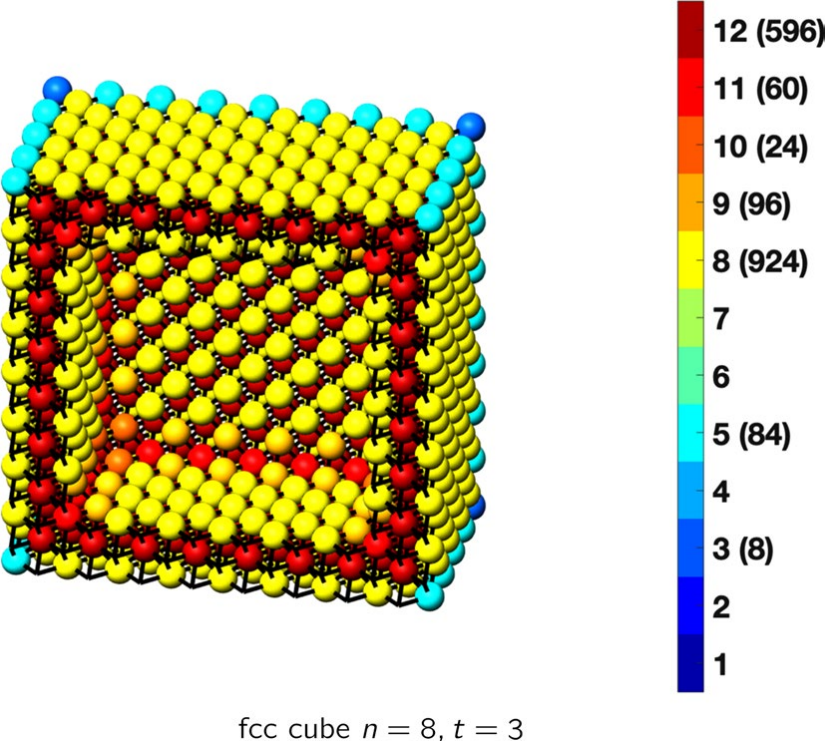

fcc cube	
Atoms	$(12t)n^2+(-12t^2+12t)n+(4t^3-6t^2+3t+1), \,n>t\ge 3$
Bonds	$(72t-48)n^2+(-72t^2+120t-36)n+(24t^3-60t^2+36t),\,n>t\ge 3$
cn = 3	$8,\,n>t\ge 3$
cn = 5	$12n-12,\,n>t\ge 3$
cn = 8	$24n^2+(-24t-24)n+(12t^2+12t+12),\,n>t\ge 3$
cn = 9	$24n +(-24t-24),\,n>t\ge 3$
cn = 10	$24,\,n>t\ge 3$
cn = 11	$12n+(-12t),\,n>t\ge 3$
cn = 12	$(12t-24)n^2+(-12t^2+36t-24)n+(4t^3-18t^2+27t-7),\,n>t\ge 3$

**Table 4:** Magic formulas for the fcc truncated cube (even layers)
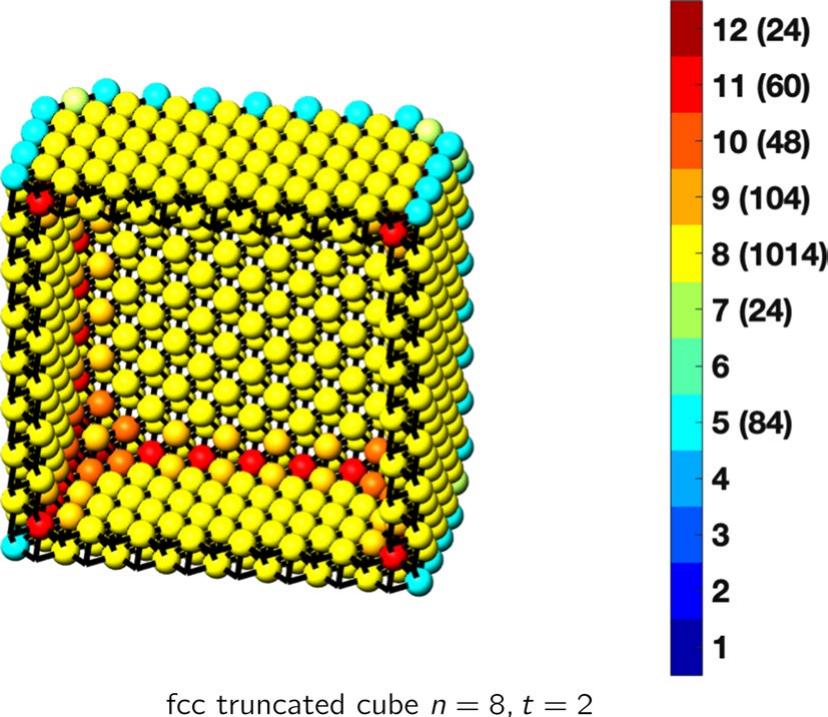

fcc truncated cube	
Atoms	$(12t)n^2+(-12t^2+12t)n+(4t^3-6t^2+3t), \,n>t\ge 2$
Bonds	$(72t-48)n^2+(-72t^2+120t-36)n+(24t^3-60t^2+36t+36),\,n>t\ge 2$
cn = 5	$12n-12,\,n>t\ge 2$
cn = 7	$24,\,n>t\ge 2$
cn = 8	$24n^2+(-24t-24)n+(12t^2+12t-18),\,n>t\ge 2$
cn = 9	$24n +(-24t -40),\,n>t\ge 2$
cn = 10	$48,\,n>t\ge 2$
cn = 11	$12n+(-12t-12),\,n>t\ge 2$
cn = 12	$(12t-24)n^2+(-12t^2+36t-24)n+(4t^3-18t^2+27t+10),\,n>t\ge 2$

**Table 5:** Magic formulas for the fcc truncated cube (odd layers)
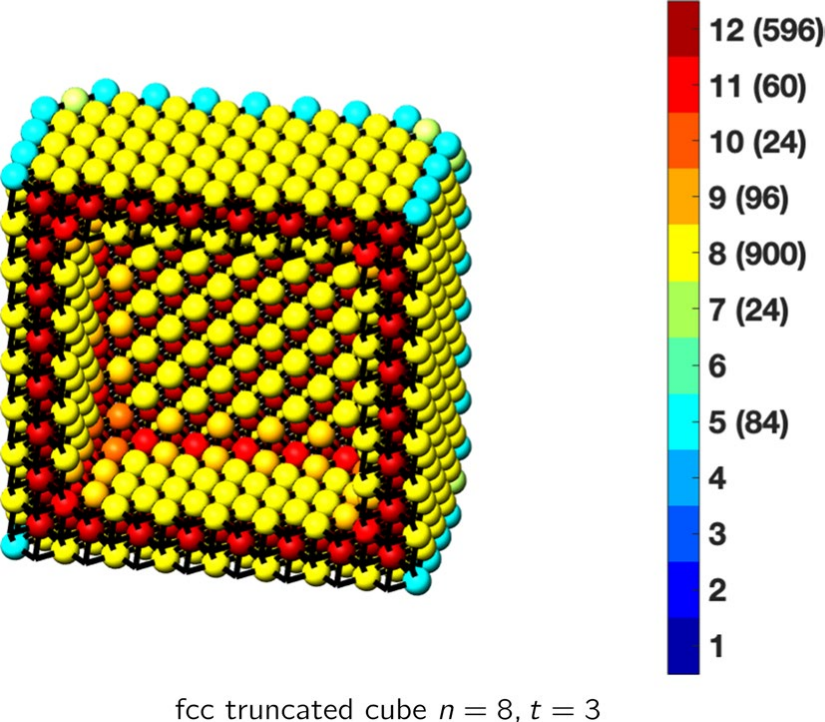

fcc truncated cube	
Atoms	$(12t)n^2+(-12t^2+12t)n+(4t^3-6t^2+3t-7), \,n>t\ge 3$
Bonds	$(72t-48)n^2+(-72t^2+120t-36)n+(24t^3-60t^2+36t-24),\,n>t\ge 3$
cn = 5	$12n-12,\,n>t\ge 3$
cn = 7	$24,\,n>t\ge 3$
cn = 8	$24n^2+(-24t-24)n+(12t^2+12t-12),\,n>t\ge 3$
cn = 9	$24n +(-24t -24),\,n>t\ge 3$
cn = 10	$24,\,n>t\ge 3$
cn = 11	$12n+(-12t),\,n>t\ge 3$
cn = 12	$(12t-24)n^2 +(-12t^2+36t-24)n+(4t^3-18t^2+27t-7),\,n>t\ge 3$

**Table 6:** Magic formulas for the fcc cuboctahedron
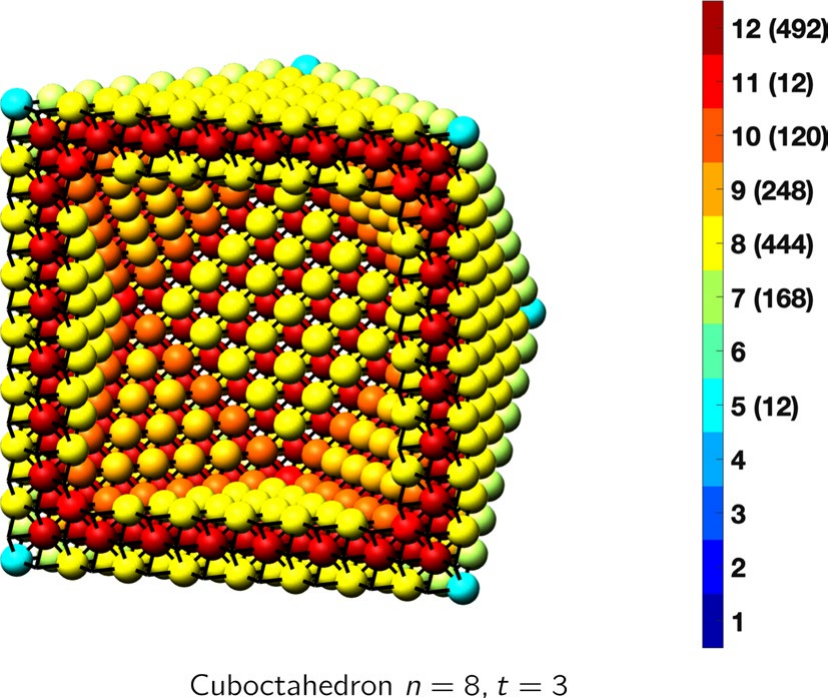

Cuboctahedron	
Atoms	$(10t)n^2+(-10t^2+10t)n+(\frac{10}{3}t^3-5t^2+\frac{11}{3}t), \,n>t\ge 3$
Bonds	$(60t-36)n^2+(-60t^2+96t-36)n+(20t^3-48t^2+40t-12),\,n>t\ge 3$
cn = 5	$12,\,n>t\ge 2$
cn = 7	$24n-24,\,n>t\ge 2$
cn = 8	$12n^2+(-12t-12)n +(6t^2+6),\,n>t\ge 2$
cn = 9	$8n^2 +(-8t-16)n+(4t^2+4t+8),\,n>t\ge 2$
cn = 10	$24n+(-24t),\,n>t\ge 2$
cn = 11	$12,\,n>t\ge 2$
cn = 12	$(10t-20)n^2 +(-10t^2+30t-20)n+(\frac{10}{3}t^3-15t^2+\frac{71}{3}t-14),\,n>t\ge 3$

**Table 7:** Magic formulas for the fcc octahedron
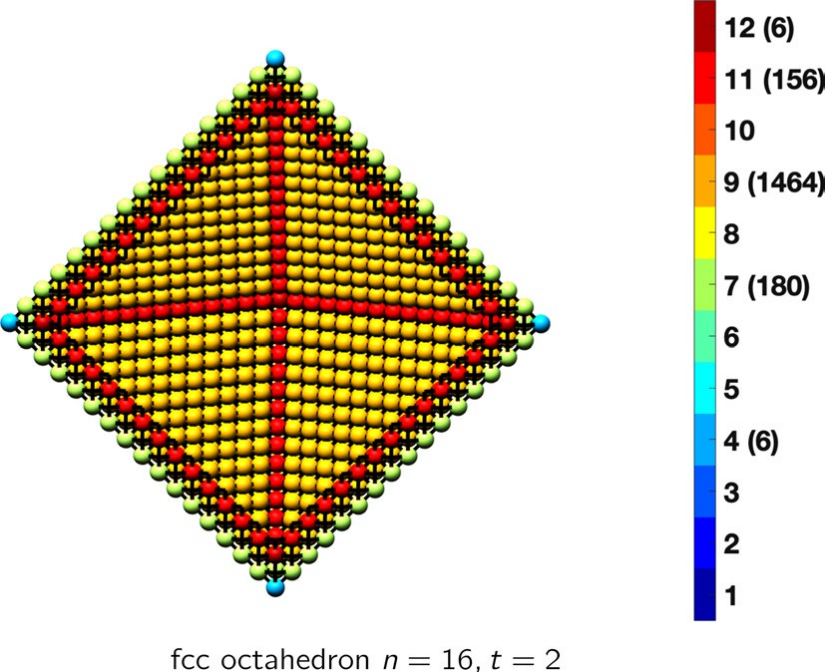

fcc octahedron	
Atoms	$(4t)n^2+(-8t^2+8t)n+(\frac{16}{3}t^3-8t^2+\frac{14}{3}t), \,n>t\ge 2$
Bonds	$(24t-12)n^2+(-48t^2+72t-24)n+(32t^3-72t^2+52t-12),\,n>t\ge 2$
cn = 4	$6,\,n>t\ge 2$
cn = 7	$12n-12,\,n>t\ge 2$
cn = 9	$8n^2+(-16t-8)n+(16t^2-8t+8),\,n>t\ge 2$
cn = 11	$12n +(-24t +12),\,n>t\ge 2$
cn = 12	$(4t-8)n^2 +(-8t^2+24t-16)n+(\frac{16}{3}t^3-24t^2+\frac{110}{3}t-14),\,n>t\ge 2$

**Table 8:** Magic formulas for the fcc tetrahedron
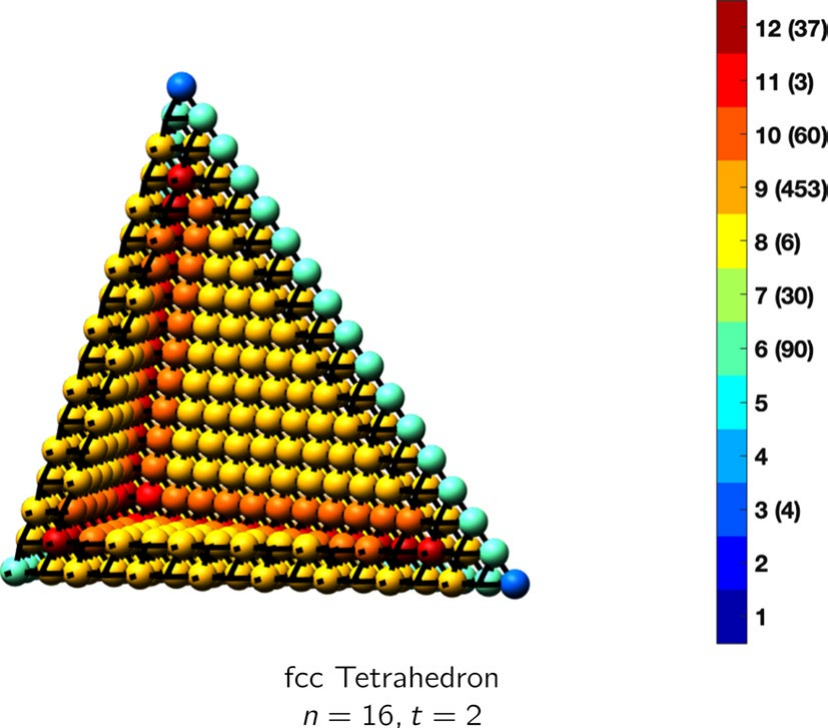

fcc tetrahedron	
Atoms	$(\frac{3}{2}t)n^2+(-\frac{9}{2}t^2+6t)n+(\frac{9}{2}t^3-9t^2+\frac{11}{2}t), \,n>t\ge 2$
Bonds	$(9t-\frac{9}{2})n^2+(-27t^2+45t-\frac{27}{2})n+(27t^3-\frac{135}{2}t^2+\frac{93}{2}t-9),\,n>t\ge 2$
cn = 3	$4,\,n>t\ge 2$
cn = 6	$6n-6,\,n>t\ge 2$
cn = 7	$3n-9t,\,n>t\ge 2$
cn = 8	$6,\,n>t\ge 2$
cn = 9	$3n^2+(-6t-12)n + (9t^2 +18t-3),\,n>t\ge 2$
cn = 10	$6n-18t,\,n>t\ge 2$
cn = 11	$3,\,n>t\ge 2$
cn = 12	$(\frac{3}{2}t-3)n^2 +(-\frac{9}{2}t^2+12t-3)n+(\frac{9}{2}t^3-18t^2+\frac{29}{2}t-4),\,n>t\ge 2$

## Icosahedral and Decahedral Nanoboxes

See Tables 9 and 10.

**Table 9:** Magic formulas for the icosahedron
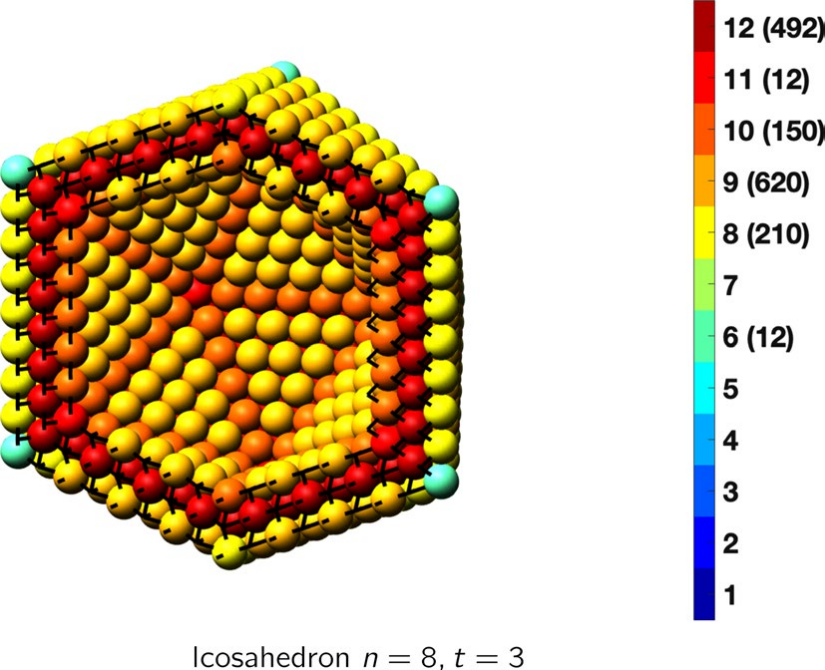

Icosahedron	
Atoms	$(10t)n^2+(-10t^2+10t)n+(\frac{10}{3}t^3-5t^2+\frac{11}{3}t), \,n>t\ge 3$
Bonds	$(60t-30)n^2+(-60t^2+90t-30)n+(20t^3-45t^2+37t-12),\,n>t\ge 3$
cn = 6	$12,\,n>t\ge 2$
cn = 8	$30n-30,\,n>t\ge 2$
cn = 9	$20n^2+(-20t-40)n +(10t^2+10t+20),\,n>t\ge 2$
cn = 10	$30n+(-30t),\,n>t\ge 2$
cn = 11	$12,\,n>t\ge 2$
cn = 12	$(10t-20)n^2 +(-10t^2+30t-20)n+(\frac{10}{3}t^3-15t^2+\frac{71}{3}t-14),\,n>t\ge 3$

**Table 10:** Magic formulas for the decahedron
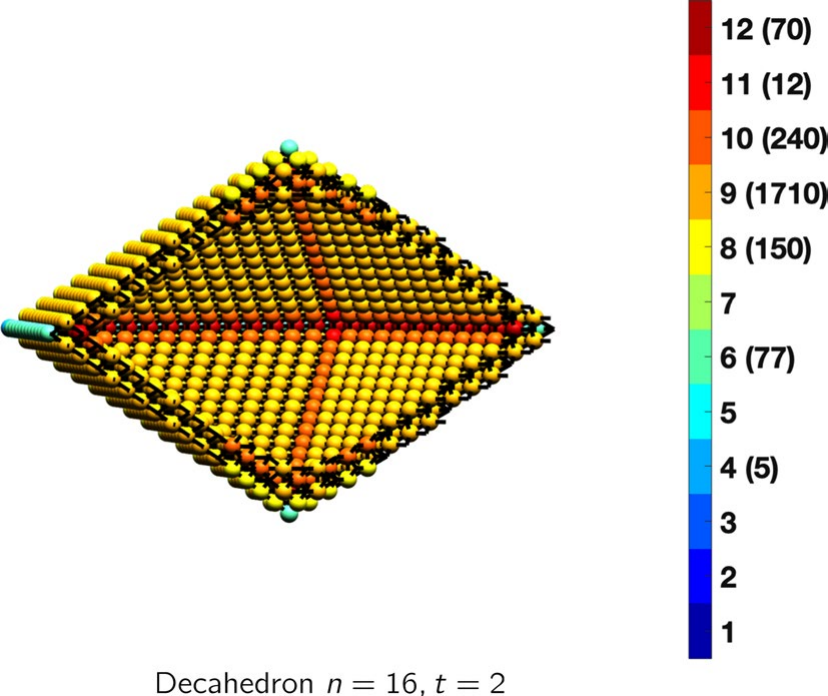

Decahedron	
Atoms	$(5t)n^2+(-10t^2+10t)n+(\frac{20}{3}t^3-10t^2+\frac{16}{3}t), \,n>t\ge 2$
Bonds	$(30t-15)n^2+(-60t^2+90t-25)n+(40t^3-90t^2+57t-12),\,n>t\ge 2$
cn = 4	$5,\,n>t\ge 2$
cn = 6	$5n-3,\,n>t\ge 2$
cn = 8	$10n-10,\,n>t\ge 2$
cn = 9	$10n^2+(-20t-20)n + (20t^2+10t+10),\,n>t\ge 2$
cn = 10	$20n+(-40t),\,n>t\ge 2$
cn = 11	$12,\,n>t\ge 2$
cn = 12	$(5t-10)n^2 +(-10t^2+30t-15)n+(\frac{20}{3}t^3-30t^2+\frac{106}{3}t-14),\,n>t\ge 2$

## Diamond and Simple Cubic Nanoboxes

The diamond cubic lattice structure is formed by an allotrope of carbon as well as the elements silicon and germanium. Also, some cubic compounds form this structure, as cubic iron oxide, tetrahedral diamond maghemite $\gamma$-Fe_2_O_3_. The bond length for Fe–O in tetrahedral diamond maghemite $\gamma$-Fe_2_O_3_ = 0.186 nm [32]. This leads to the diameter of diamond clusters *D*(*n*) as below:

16 $$D(n) = 3.3984\cdot n_{{\rm B}} \cdot n - 0.21194.$$

According to reference [12], microboxes of cubic iron oxide formed and had interesting lithium storage capabilities. We are not aware of a complete coordination model for energy storage, but as mentioned above, DFT results indicate that activity may depend on facet orientation [19]. No such model of storage dependence on coordination exists presently as we have for catalysis. From equation (16) above (created using *t* = 4), a microbox requires approximately *n* = 1600 shells for diamond maghemite. Magical formulas for the diamond and simple cubic lattice structures are listed below (Tables 11, 12).

**Table 11:** Magic formulas for diamond nanoboxes
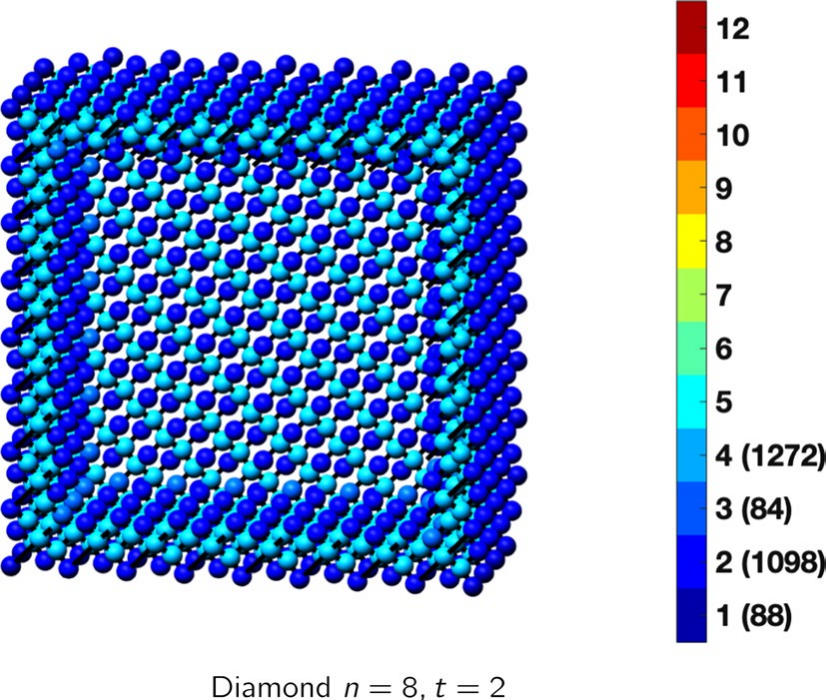

Diamond (only even *t* allowed)	
Atoms	$(24t)n^2+(-24t^2+12t)n+(8t^3-6t^2+3t), \,n>t\ge 2$
Bonds	$(48t-24)n^2+(-48t^2+48t-12)n+(16t^3-24t^2+12t+12),\,n>t\ge 2$
cn = 1	$12n-8,\,n>t\ge 2$
cn = 2	$24n^2+(-24t-12)n+(12t^2-6),\,n>t\ge 2$
cn = 3	$12n+(-12t+12),\,n>t\ge 2$
cn = 4	$(24t-24)n^2 +(-24t^2+36t-12)n+(8t^3-18t^2+15t+2),\,n>t\ge 2$

**Table 12:** Magic formulas for the simple cube
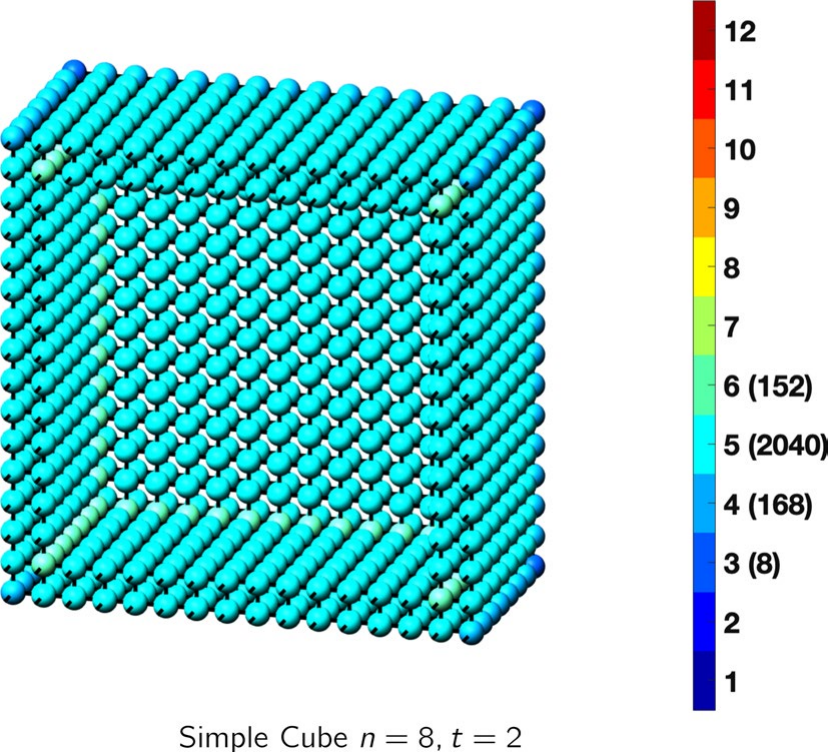

Simple cube	
Atoms	$(24t)n^2+(-24t^2)n+(8t^3), \,n>t\ge 2$
Bonds	$(72t-24)n^2+(-72t^2+24t)n+(24t^3-12t^2),\,n>t\ge 2$
cn = 3	$8,\,n>t\ge 2$
cn = 4	$24n-24,\,n>t\ge 2$
cn = 5	$48n^2+(-48t-48)n + (24t^2+24),\,n>t\ge 2$
cn = 6	$(24t-48)n^2 +(-24t^2+48t+24)n+(8t^3-24t^2-8),\,n>t\ge 2$

## BCC Nanoboxes

See Tables 13, 14 and 15.

**Table 13:** Magic formulas for the bcc cube
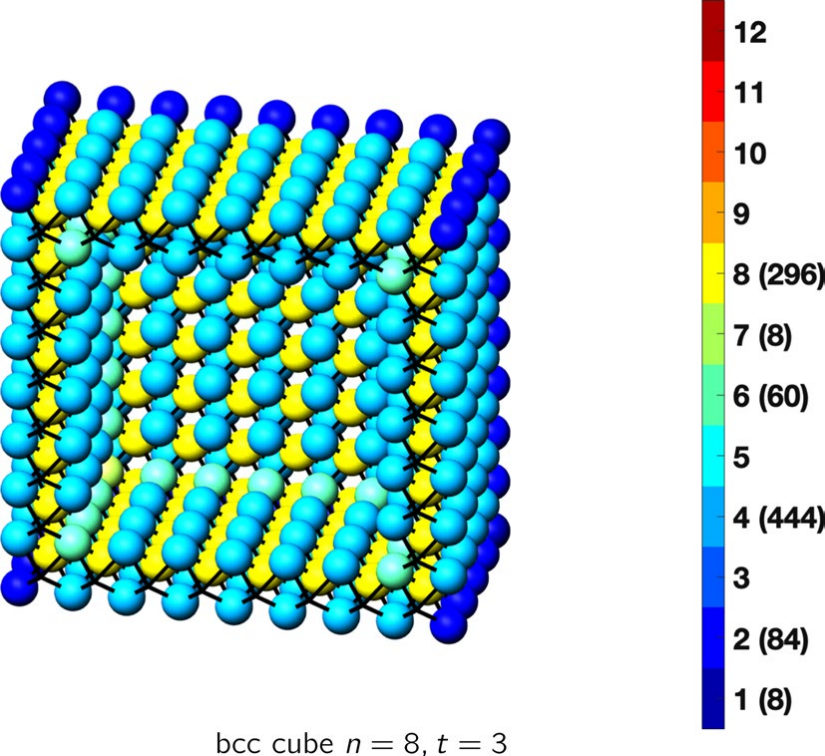

bcc cube	
Atoms	$(6t)n^2+(-6t^2+6t)n+(2t^3-3t^2+3t), \,n>t\ge 3$
Bonds	$(24t-24)n^2+(-24t^2+48t-24)n+(8t^3-24t^2+24t-8),\,n>t\ge 3$
cn = 1	$8,\,n>t\ge 2$
cn = 2	$12n-12,\,n>t\ge 2$
cn = 4	$12n^2 + (-12t-12)n +(6t^2+6),\,n>t\ge 2$
cn = 6	$12n +(-12t),\,n>t\ge 2$
cn = 7	$8,\,n>t\ge 2$
cn = 8	$(6t-12)n^2 +(-6t^2+18t-12)n+(2t^3-9t^2+15t-10),\,n>t\ge 3$

**Table 14:** Magic formulas for the bcc truncated cube
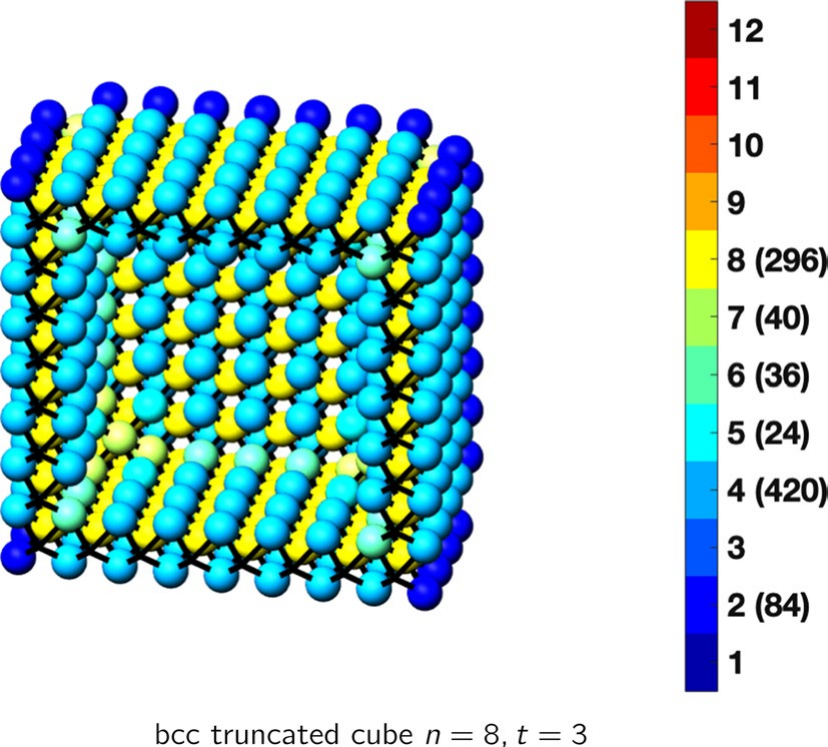

bcc truncated cube	
Atoms	$(6t)n^2+(-6t^2+6t)n+(2t^3-3t^2+3t), \,n>t\ge 3$
Bonds	$(24t-24)n^2+(-24t^2+48t-24)n+(8t^3-24t^2+24t+40),\,n>t\ge 3$
cn = 2	$12n-12,\,n>t\ge 2$
cn = 4	$12n^2 + (-12t-12)n +6t^2-18,\,n>t\ge 2$
cn = 5	$24,\,n>t\ge 2$
cn = 6	$12n +(-12t -24),\,n>t\ge 2$
cn = 7	$40,\,n>t\ge 2$
cn = 8	$(6t-12)n^2 +(-6t^2+18t-12)n+(2t^3-9t^2+15t-10),\,n>t\ge 3$

**Table 15:** Magic formulas for the bcc rhombic dodecahedron
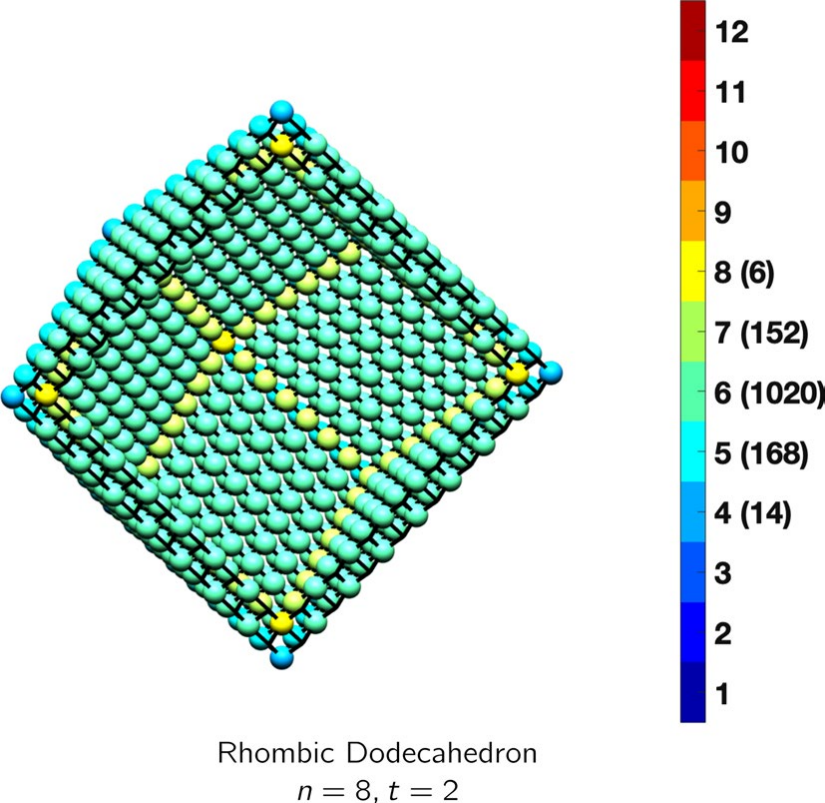

bcc rhombic dodecahedron	
Atoms	$(12t)n^2+(-12t^2+12t)n+(4t^3-6t^2+4t), \,n>t\ge 2$
Bonds	$(48t-24)n^2+(-48t^2+72t-24)n+(16t^3-36t^2+28t-8),\,n>t\ge 2$
cn = 4	$14,\,n>t\ge 2$
cn = 5	$24n-24,\,n>t\ge 2$
cn = 6	$24n^2+(-24t-24)n + (12t^2+12),\,n>t\ge 2$
cn = 7	$(24)n+(-24t+8),\,n>t\ge 2$
cn = 8	$(12t-24)n^2 +(-12t^2+36t-24)n+(4t^3-18t^2+24t-10),\,n>t\ge 2$

## HCP Nanoboxes

See Table 16.

**Table 16:** Magic formulas for the hexagonal bipyramid
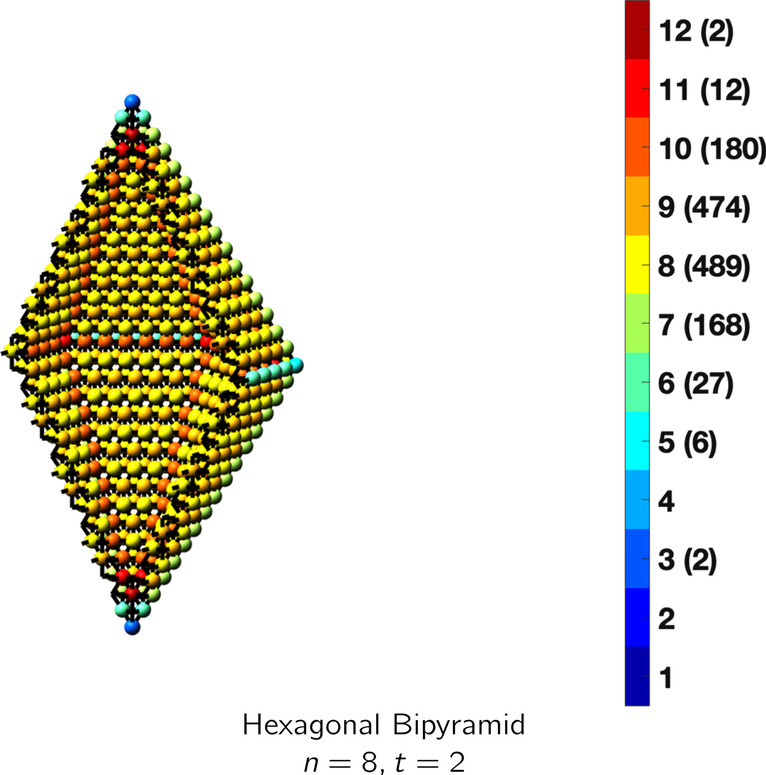

Hexagonal bipyramid	
Atoms	$(12t)n^2+(-12t^2+12t)n+(4t^3-6t^2+4t), \,n>t\ge 2$
Bonds	$(72t-42)n^2+(-72t^2+114t-42)n+(24t^3-57t^2+45t-12),\,n>t\ge 2$
cn = 3	$2,\,n>t\ge 2$
cn = 5	$6,\,n>t\ge 2$
cn = 6	$3n+3,\,n>t\ge 2$
cn = 7	$(24)n - 24,\,n>t\ge 2$
cn = 8	$12n^2+(-12t-15)n + (6t^2+9),\,n>t\ge 2$
cn = 9	$12n^2+(-12t-18)n+(6t^2+6t+6),\,n>t\ge 2$
cn = 10	$30n+(-30t),\,n>t\ge 2$
cn = 11	$12,\,n>t\ge 2$
cn = 12	$(12t-24)n^2 +(-12t^2+36t-124)n+(4t^3-18t^2+28t-14),\,n>t\ge 2$

## The Case *t* = 1

The special case *t* = 1 is unique and as such has distinct magical formulas. We examine this case for some of the above nanoboxes. Nanoboxes with ultrathin walls have been formed with cubic [33], octahedral [16], and icosahedral shapes [34]. According to the magical formulas below, the cubic nanobox with *t* = 1 has the lowest coordination. Platinum has a relatively high reduction potential of 1.18 V versus the SHE, so it can be formed by galvanic replacement, see Eq. (1) [5]. However, the oxidation reduction reaction (ORR) properties of some of these platinum-based nanocages indicate that structures with (111) facets as opposed to (100) facets have better ORR mass activities [35].

Thus the icosahedron with 20 (111) facets has the best ORR mass activity, followed by the octahedron, and lastly the truncated cube. This property of catalytic behavior from facet orientation taking precedence over coordination number is evidenced by the tabular data below. In other words, as mentioned in the following tables, the cube with (100) facets has the lowest magic coordination numbers with four and five, yet the octahedron and icosahedron with (111) facets and larger magic formulas have better ORR activity. This property is evidenced in nanoclusters as well, where DFT results confirm the dominance of the (111) facets [36], especially for PtNi alloys (Tables 17, 18, 19, 20, 21).

**Table 17:** Magic formulas for the truncated cube
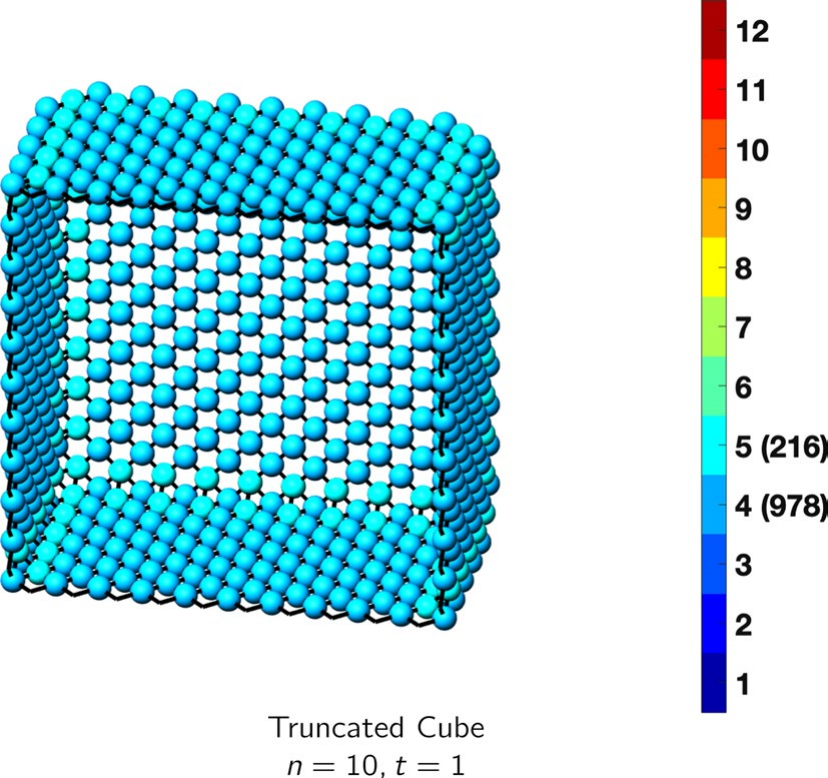

Truncated cube	
Atoms	$12n^2-6, \,n>t = 1$
Bonds	$24n^2+12n-24, \,n>t = 1$
cn = 4	$12n^2-24n+18,\,n>t = 1$
cn = 5	$24n-24,\,n>t=1$

**Table 18:** Magic formulas for the icosahedron
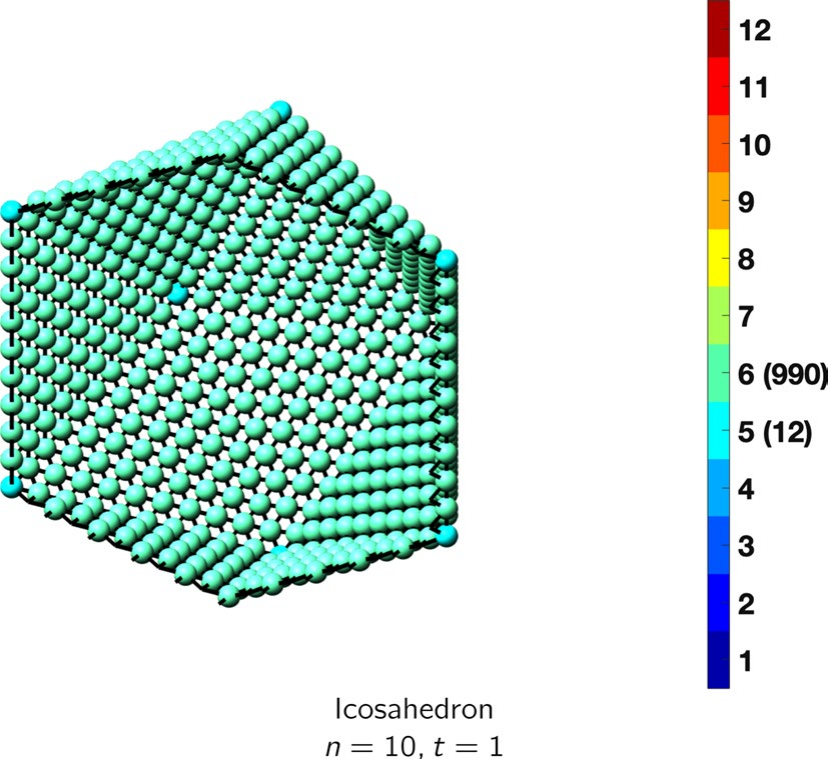

Icosahedron	
Atoms	$10n^2+2, \,n>t = 1$
Bonds	$30n^2, \,n>t = 1$
cn = 5	$12,\,n>t = 1$
cn = 6	$10n^2-10,\,n>t=1$

**Table 19:** Magic formulas for the cuboctahedron
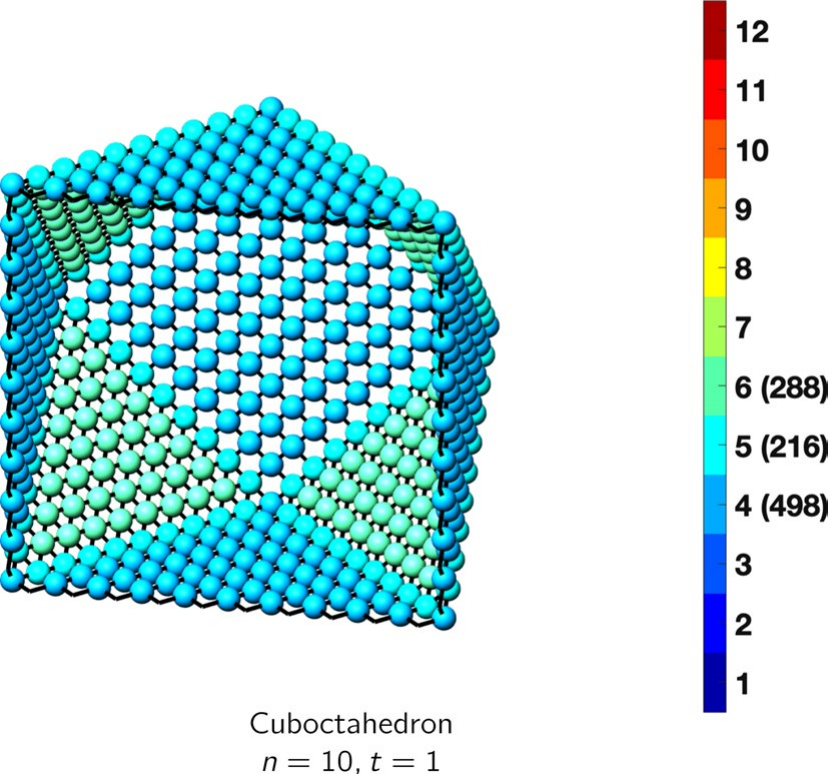

Cuboctahedron	
Atoms	$10n^2+2, \,n>t = 1$
Bonds	$24n^2, \,n>t = 1$
cn = 4	$6n^2-12n+18,\,n>t = 1$
cn = 5	$24n-24,\,n>t=1$
cn = 6	$4n^2-12n+8,\,n>t=1$

**Table 20:** Magic formulas for the octahedron
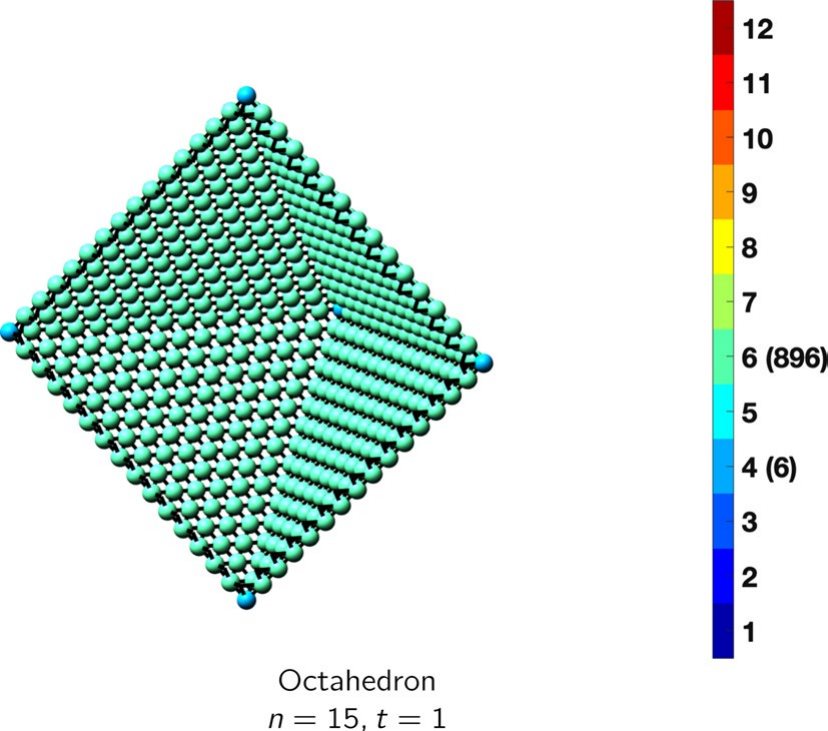

Octahedron	
Atoms	$4n^2+2, \,n>t = 1$
Bonds	$12n^2, \,n>t = 1$
cn = 4	$6,\,n>t = 1$
cn = 6	$4n^2-4,\,n>t=1$

**Table 21:** Magic formulas for the decahedron
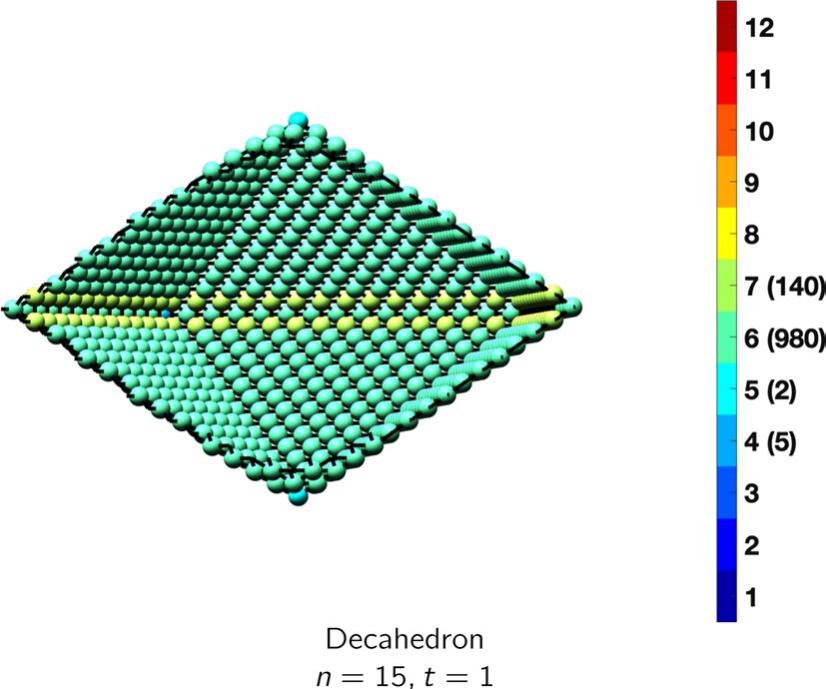

Decahedron	
Atoms	$5n^2+2, \,n>t = 1$
Bonds	$15n^2+5n-5, \,n>t = 1$
cn = 4	$5,\,n>t = 1$
cn = 5	$2,\,n>t = 1$
cn = 6	$5n^2-10n+5,\,n>t = 1$
cn = 7	$10n-10,\,n>t=1$

## Dispersion

**Fig. 1:**
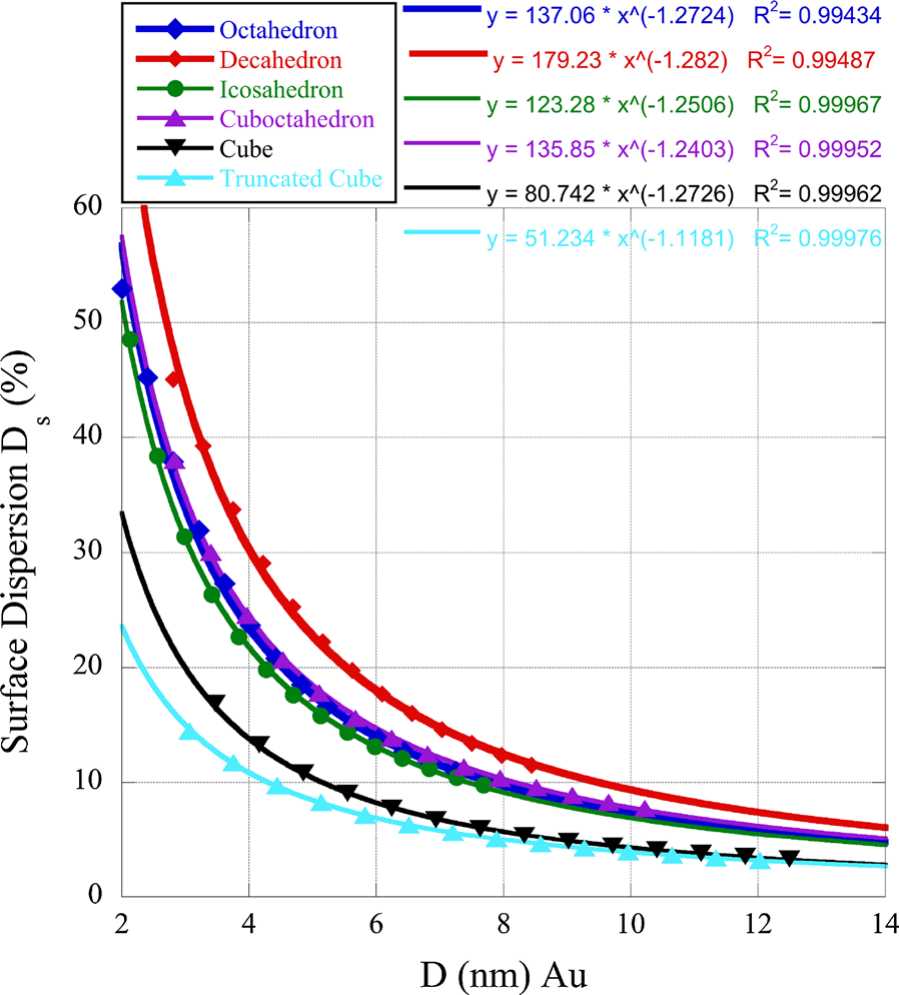
Surface dispersion of gold nanoboxes

Given the importance of edge and kink sites relative to facet ones with regard to catalytic activity, we have determined the surface dispersion for some of the nanoboxes we study. The (100) facets have cn = 8 while the (111) facets have cn = 9. This may provide insight into the reasons for the individual polyhedral activity when compared among the nanoboxes. In Fig. 1 below, we plot the surface dispersion $D_{{\rm s}} = (N_{{\rm e}} + N_{{\rm k}}) / N_{{\rm S}}\cdot 100\%$. In this relationship $N_{{\rm k}}$ is the number of kink or corner sites and $N_{{\rm e}}$ the number of edge sites. As can be seen in Figure 1, nanoboxes with (111) surfaces as opposed to (100) surfaces have higher dispersion, giving credence to the preference of catalytic activity of the (111) facet.

## Conclusion

In summary, we have presented the first detailed mathematical description of magical formulas for nanoboxes. The case of the shell thickness, *t* = 1 is distinct from $t>1$ and we tabulate the data for some of these cases. The formulas for the coordination, number of atoms, and number of bonds are all enumerated. We find that bulk coordination appears for layers where *t* = 2 or 3, and as such is much thinner than normally synthesized. The benefits of low coordination are only achieved for very thin walls. We expect these results to be useful for modeling and experimental work.

## Data Availability

The dataset(s) supporting the conclusions of this article may be obtained from the corresponding author.

## Abbreviations

bcc: Body centered cubic
fcc: Face centered cubic
hcp: Hexagonal close packed
DFT: Density functional theory
SHE: Secondary hydrogen electrode
